# Time until treatment initiation is associated with catheter survival in peritoneal dialysis-related peritonitis

**DOI:** 10.1038/s41598-021-86071-y

**Published:** 2021-03-22

**Authors:** Rikako Oki, Shiho Tsuji, Yoshifumi Hamasaki, Yohei Komaru, Yoshihisa Miyamoto, Ryo Matsuura, Daisuke Yamada, Kent Doi, Haruki Kume, Masaomi Nangaku

**Affiliations:** 1 Department of Hemodialysis and Apheresis, The University of Tokyo Hospital, 7-3-1 Hongo, Bunkyo-ku, Tokyo 113-8655 Japan; 2 Department of Acute Medicine, The University of Tokyo Hospital, Bunkyo-ku, Japan; 3 Department of Urology, The University of Tokyo Hospital, Bunkyo-ku, Japan

**Keywords:** Renal replacement therapy, Peritoneal dialysis, Risk factors

## Abstract

For peritonitis, a serious complication of peritoneal dialysis (PD), we investigated the relation between duration from the sign (PD effluent abnormalities) to treatment with appropriate antibiotics (ST time) and catheter removal. For 62 PD hospital patients, data of PD-related peritonitis (*n* = 109) were collected retrospectively. We examined ST time and PD catheter removal times using univariate and multivariate analyses. The catheter removal rate in the delayed ST time group (≥ 24 h) was higher than that in early ST time group (< 24 h) (38 vs. 16%, *p* = 0.02). Concomitant tunnel infection and delayed ST time were associated with catheter removal (OR [95% CI] 32.3 [3.15–329] and 3.52 [1.11–11.1]). Rates of catheter removal and re-development of peritonitis within 1 month after starting treatment were higher in the delayed ST time group (*p* = 0.02). PD duration at peritonitis and the first peritonitis episode were associated with delayed ST time (1.02 [1.00–1.04] and 3.42 [1.09–10.7]). Significant association was found between PD catheter removal and the start of treatment more than 24 h after appearance of abnormal effluent. Education for patients about prompt visitation at the onset of peritonitis with long PD duration might improve outcomes.

## Introduction

Although peritoneal dialysis (PD) is described as an effective treatment modality for patients with end-stage renal disease, peritonitis remains a major complication of PD, which might result in discontinuation of PD^1^. A NEXT-PD study revealed that peritonitis accounted for 17.6% of all causes of PD discontinuation^2^. The mortality risk ascribed to PD-related peritonitis is reported as higher than 15% in PD patients^3^. As described in ISPD guidelines, catheter removal must be considered when no clinical improvement occurs within 5 days while continuing appropriate antibiotic treatment^1^. Delayed or insufficient treatment for peritonitis might require removal of a PD catheter and subsequent transition to hemodialysis^1^. Choi reported that more than half of patients with peritonitis who underwent catheter removal also experienced postoperative hospitalization longer than 10 days^4^. Moreover, only 4% of patients after PD catheter removal were able to have re-insertion and continue long-term PD therapy^4^. Because this surgical procedure is burdensome for patients, it seems desirable that peritonitis be treated with appropriate conservative therapy to avoid unnecessary catheter loss. Therefore, elucidating risk factors for catheter removal is necessary to choose a therapeutic strategy against PD-related peritonitis promptly and properly.

PD-related peritonitis is characterized by cloudy PD effluent and abdominal pain^1^. Some patients present only cloudy effluent, without abdominal pain. Because prompt initiation of antibiotics treatment is crucially important for peritonitis, the International Society for Peritoneal Dialysis (ISPD) has recommended that patients with suspected peritonitis who live far from medical facilities should receive administration of antibiotics at a nearby hospital^1^. Consequently, patients at our hospital are educated to have access to the hospital immediately after awareness of the cloudy PD effluent. Although certain consensus has been established for the importance of early initiation of antibiotics for peritonitis, little is known about whether the early start of treatment can decrease the risk of catheter removal.

This study was conducted to investigate the role of prompt treatment for PD-related peritonitis by examining the relation between the time from awareness of abnormalities of effluent to the initiation of treatment and catheter removal. We also examined factors related to the delay of starting appropriate treatment for PD-related peritonitis.

## Results

**Figure 1:**
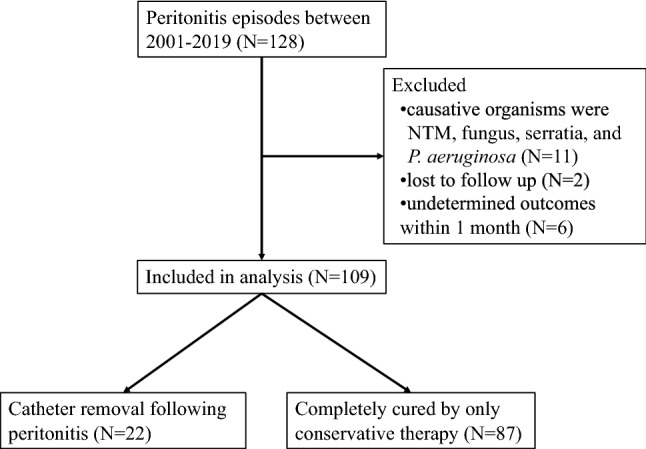
Chart showing flow of the study.

During the study period, 159 end-stage renal disease (ESRD) patients initiated PD at our hospital as their first renal replacement therapy. Of them, 66 developed 128 cases of PD-related peritonitis. The averaged peritonitis incidence was 0.22 per patient-year in our facility. After exclusion of 19 peritonitis cases for which PD catheter removal was decided based on the type of causative organism or for which PD catheter survival could not be followed-up, 109 peritonitis cases of 62 patients were finally examined as study subjects (Fig. 1). Of 62 patients, 52 (84%) were male, with primary diseases of ESRD including diabetic nephropathy (*n* = 26, 42%), chronic glomerulonephritis (*n* = 20, 32%), nephrosclerosis (*n* = 9, 15%), and others (*n* = 7, 11%).

Table 1 presents the characteristics of 109 peritonitis cases. Mean patient age at peritonitis onset was 62.9 ± 13.3. The median PD duration at peritonitis onset was 29.6 (10.5, 49.0) months. Automated PD and PD + HD combination therapy were administered respectively in 78% and 35% of the peritonitis cases. PD catheter was removed in 22 cases (20%). Only 1 patient in ST time < 24 h group died, because of peritonitis. The median ST time was 6.0 (4.0, 17.7) hours.

**Table 1:** Comparison between removal and non-removal groups.

	All (*N* = 109)	Removal group (*N* = 22)	Non-removal group (*N* = 87)	*p* (removal vs. non-removal)
Age at peritonitis [year old]	62.9 ± 13.3	62.4 ± 12.7	63.1 ± 13.5	0.83
DM	53 (49%)	10 (45%)	43 (49%)	0.74
PD duration at peritonitis [months]	29.6 (10.5, 49.0)	37.6 (17.0, 69.5)	27.6 (8.67, 47.6)	0.02
Use of automated PD devices at Peritonitis [*n*(%)]	99 (91%)	18 (82%)	81 (93%)	0.10
PD + HD combination therapy at peritonitis [*n*(%)]	38 (35%)	10 (45%)	28 (32%)	0.24
First peritonitis episode	60 (55%)	13 (59%)	47 (54%)	0.67
Use of oral antibiotics before visiting hospital	20 (18%)	6 (27%)	14 (16%)	0.23
Concomitant tunnel infection	5 (5%)	4 (18%)	1 (1%)	< 0.01
Abdominal pain at presentation	49 (47%)	11 (52%)	38 (46%)	0.59
WBC in PD fluid at presentation [/μl]	2300 (850, 5150)	2250 (800, 4825)	2500 (950, 6350)	0.93
ST time	6.0 (4.0, 17.7)	8.95 (3.75, 39.3)	5.5 (4.0, 12.5)	0.13
ST time ≥ 24 h [*n*(%)]	21 (19%)	8 (36%)	13 (15%)	0.02
Time from treatment initiation to catheter removal [day]	–	6.5 (4.0, 12.3)	–	–

Continuous data are presented as median (IQR): *DM* diabetes mellitus, *PD* peritoneal dialysis, *HD* hemodialysis, *WBC* white blood cell, *ST* time, required time from sign to treatment initiation.

The most common causative organism of peritonitis was *Streptococcus* spp. (16%), followed by Coagulase negative *Staphylococcus* (CNS) (15%), gram-negative bacilli (13%), and *Staphylococcus aureus* (9%). Culture negative peritonitis was 42 cases (39%). There was no difference in causative microorganism between removal and non-removal groups/ST time more than 24 h and less than 24 h groups (see Supplementary Tables 1, 2). Intravenous and/or intraperitoneal antibiotic administration was performed in all peritonitis cases.

Table 1 presents results of comparison of variables between two groups: with and without PD catheter removal. Significant difference was found in the rate of patients with long ST time (≥ 24 h) between two groups (36% in the removal group vs. 15% in the non-removal group, *p* = 0.02). Duration from initiation of intraperitoneal/intravenous antibiotics treatment to PD catheter removal was 6.5 days. The rate of concomitant tunnel infection was significantly higher in the removal group (18% vs. 1%, *p* < 0.01).

**Figure 2:**
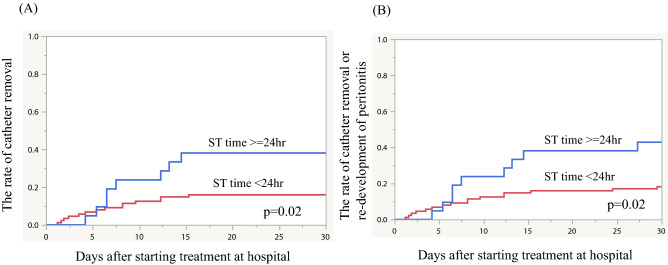
Kaplan–Meier analysis for the rate of catheter removal and relapse/recurrence of peritonitis during 1 month from initiation of treatment. Rate of catheter removal (**A**) and a composite outcome of catheter removal or relapse/recurrence of peritonitis (**B**) during 1 month from initiation of treatment were significantly higher in the ST time ≥ 24 h group compared with the ST time < 24 h group (*p* = 0.02, both).

Table 2 presents results of univariate and multivariate logistic regression analysis for PD catheter removal within 1 month after starting treatment at hospital. These results revealed that concomitant tunnel infection and ST time ≥ 24 h were independent predictors of PD catheter removal following peritonitis (OR [95% CI] 32.2 [3.15–329] and 3.52 [1.11–11.1], respectively). The PD catheter survival rates during 1 month after the start of treatment were compared between groups with ST time < 24 and ≥ 24 h using Kaplan–Meier analysis (Fig. 2A). Results showed that the PD catheter removal rate in the group with ST time ≥ 24 h was significantly higher than that with ST time < 24 h group (*p* = 0.02, Fig. 2A). When the rate of PD catheter survival or re-development of peritonitis within 1 month after initiation of treatment was also examined, similar results were obtained (*p* = 0.02, Fig. 2B).

**Table 2:** Results of univariate and multivariate logistic regression analysis for catheter removal.

Variables	Univariate		Multivariate	
	OR (95% CI)	*p*	OR (95% CI)	*p*
Age at peritonitis	1.00 (0.96–1.03)	0.83		
DM	0.85 (0.33–2.18)	0.74		
PD duration at peritonitis	1.02 (1.00–1.04)	0.02	1.02 (1.00–1.04)	0.06
Use of automated PD devices at peritonitis	0.33 (0.09–1.30)	0.13		
PD + HD combination therapy at peritonitis	1.76 (0.68–4.55)	0.25		
First peritonitis episode	1.23 (0.48–3.17)	0.67		
Use of oral antibiotics before visiting hospital	1.96 (0.65–5.87)	0.24		
Concomitant tunnel infection	19.1 (2.02–181)	< 0.01	32.2 (3.15–329)	< 0.01
Abdominal pain at presentation	1.30 (0.50–3.40)	0.59		
WBC in PD fluid at presentation	1.0 (1.00–1.00)	0.93		
ST time	1.01 (1.00–1.02)	0.16		
ST time ≥ 24 h	3.25 (1.14–9.30)	0.03	3.52 (1.11–11.1)	0.03

Table 3 presents a comparison of the values found for variables for groups with ST time < 24 h (early group) and ≥ 24 h (late group). The PD catheter removal rate was higher in the longer ST time group (38% vs. 16%). The PD duration at peritonitis was significantly longer in the late group than in the early group. Patients in the late group had more first peritonitis episodes and were more likely to use oral antibiotics before visiting a hospital. Multivariate logistic regression analysis showed that the first peritonitis episode and longer PD duration were associated with long ST time (≥ 24 h) (OR [95% CI] 3.42 [1.09–10.7] and 1.02 [1.00–1.04], respectively) (Table 4).

**Table 3:** Comparison of groups with ST time more or less than 24 h.

	ST time < 24 h (*N* = 88)	ST time ≥ 24 h (*N* = 21)	*p*
Age at peritonitis [years old]	63.2 ± 13.8	61.6 ± 11.2	0.62
DM	44 (50%)	9 (43%)	0.56
PD duration at peritonitis [months]	26.5 (8.8, 47.6)	40.7 (16.3, 70.0)	0.01
Use of automated PD devices at peritonitis [*n*(%)]	80 (91%)	19 (90%)	0.95
PD + HD combination therapy at peritonitis [*n*(%)]	32 (36%)	6 (29%)	0.50
First peritonitis episode	44 (50%)	16 (76%)	0.03
Use of oral antibiotics before visiting hospital	12 (14%)	8 (38%)	< 0.01
Concomitant tunnel infection	5 (6%)	0 (0%)	0.26
Abdominal pain at presentation	41 (49%)	8 (38%)	0.35
WBC in PD fluid at presentation [/μl]	2350 (900, 4975)	1400 (700, 5800)	0.87
PD catheter removal	14 (16%)	8 (38%)	0.02
Distance from patients’ residence to the hospital [km]	10.46 (5.05, 26.5)	10.83 (8.50, 16.0)	0.64

**Table 4:** Results of univariate and multivariate logistic regression analysis for ST time ≥ 24 h.

Variable	Univariate		Multivariate	
	OR (95% CI)	*p*	OR (95% CI)	*p*
Age at peritonitis	0.99 (0.95–1.03)	0.61		
DM	0.75 (0.29–1.96)	0.56		
PD duration at peritonitis	1.02 (1.00–1.04)	0.02	1.02 (1.00–1.04)	0.04
Use of automated PD devices at peritonitis [*n*(%)]	0.95 (0.19–4.84)	0.95		
PD + HD combination therapy at peritonitis [*n*(%)]	0.70 (0.25–1.98)	0.50		
tHe first peritonitis episode	3.20 (1.08–9.50)	0.03	3.42 (1.09–10.7)	0.04
Use of oral antibiotics before visiting hospital	3.90 (1.34–11.4)	0.02	2.68 (0.83–8.71)	0.11
Concomitant tunnel infection	3.63e−7 (0.0–0.0)	0.99		
Abdominal pain at presentation	0.63 (0.24–1.68)	0.36		
WBC in PD fluid at presentation [/μl]	1.00 (1.00–1.00)	0.86		
Distance from patients’ residence to the hospital [km]	1.00 (0.99–1.02)	0.65		

We also compared the catheter removal and re-development of peritonitis rates of the patient groups divided by ST times of 12 h and 18 h, respectively (Supplementary Table 3). No significant difference was found in the catheter removal rates between the two groups divided by ST time of 12 h. Univariate logistic regression analysis revealed that ST time ≥ 12 h was not related significantly to catheter removal (OR [95%CI] 1.93 [0.73–5.10], *p* = 0.19). However, significant difference was found when the cutoff of ST time was set to 18 h, although univariate logistic regression analysis showed that ST time ≥ 18 h was not related significantly to catheter removal (OR [95%CI]: 2.65 [0.98–7.18], *p* = 0.06) (Supplementary Table 4). When ST time was used as a continuous variable, it was found not to be correlated significantly with the catheter removal rate (Table 2).

From Kaplan–Meier analysis results, no significant difference in the PD catheter removal rate was found between the groups with ST time ≥ 12 h and < 12 h (*p* = 0.21). (Supplementary Fig. 1) Similar results were obtained when setting the cut-off value of ST time to 18 h (*p* = 0.08). The rate of PD catheter survival or re-development of peritonitis within 1 month after initiation of treatment was significantly higher in the ST time ≥ 18 h group than that with ST time < 18 h group (*p* = 0.01) (*p* = 0.01, Supplementary Fig. 2B).

## Discussion

For this retrospective observational cohort study, we analyzed 109 peritonitis cases occurring in 62 PD patients to examine factors related to catheter removal. Few studies have specifically examined the relation between the actual required time from awareness of cloudy effluent to initiation of treatment and catheter loss. Our results demonstrated that concomitant tunnel infection and ST time > 24 h were associated significantly with PD catheter removal because of peritonitis. In addition, the PD duration at peritonitis and the first peritonitis episode were detected as independent factors related to ST time longer than 24 h. Although ST time ≥ 24 h was found to be associated significantly with PD catheter removal, no association was found between ST time and catheter removal when the ST time was divided at 12 h or 18 h. Several possible reasons for this result can be considered. First, because information about the time of awareness of the cloudy effluent was obtained from medical record entries based on patient interviews at the time of emergency visit, some difference might exist between the actual time and the patient-reported time. Therefore, if the ST time range setting becomes narrower, then the discrepancy between the actual and the obtained ST time can be larger. Second, because most automated peritoneal dialysis (APD) patients at our facility observe their PD effluent in the morning of each day, the time from the true onset of peritonitis to awareness of cloudy effluent might be long, unlike continuous ambulatory peritoneal dialysis (CAPD) patients, who change their bags every few hours throughout the day. As more than 90% of patients were treated by APD in our study, the impact of slight differences of ST time in the early hours on the success of peritonitis treatment might have appeared to be lessened.

As described in the ISPD guidelines, successful treatment of PD related peritonitis apparently requires the start of antibiotics administration without delay. The PROMPT study in Australia demonstrated that contact to treatment time (CT time), defined as the time between the first health provider contact and introduction of antimicrobial therapy, is independently associated with PD failure (catheter removal or death) at 30 days (OR[95%CI] 1.068 [1.01–1.13], *p* = 0.02)^5^. It is particularly interesting that the relation between CT time and PD discontinuation was significant when peritonitis patients initially presented to hospital-based facility, but not to an ambulant care facility^5^. The authors implied that presentation to hospitals with differing triage priorities or to those unfamiliar with peritonitis might cause a delay in starting antibiotic treatment. Results from our study showed that ST time ≥ 24 h is independently associated with catheter removal or death. Results suggest that the patients with suspected peritonitis according to cloudy effluent should visit a hospital as quickly as possible and that medical staff should start antibiotic treatment promptly. At medical facilities, establishing an action protocol for peritonitis and support or training for medical staff who are unfamiliar with PD will be necessary to reduce the time until starting treatment. At our hospital, because the management for all peritonitis cases is determined by the nephrologist from the initial treatment and because an action algorithm for peritonitis is established, there might be less delay in initiating antibiotic treatment after the visit. This might be one reason why the CT time was not associated with catheter loss in our study, unlike earlier reports.

Why was a longer ST time associated with the risk of PD catheter removal or death following peritonitis? As described above, ISPD guidelines recommend starting intraperitoneal antibiotics as soon as possible when a patient is presumed to have peritonitis^1^. Generally, delayed starting initial antimicrobial therapy can allow pathogens to proliferate, rendering patients less responsive to treatment. Moreover, biofilms that form because of delayed treatment initiation might contribute to increased risk of catheter removal by re-development of peritonitis after initiation of treatment. Analyzing 64 episodes of acute peritonitis requiring catheter removal, Choi et al. reported that 20% of patients required catheter removal within 6 weeks following recurrent peritonitis caused by the same organism as in the previous episode^4^. The biofilm formation mechanism is described as follows^6, 7^. (1) The immune response to the catheter itself develops the formation of a conditioning film, consisting of leukocytes, macrophages, and mesothelial cells. (2) The bacteria will be encased in multiple layers of extracellular matrix such as extracellular polymeric substances produced by themselves. (3) Inflammatory cells collect around this structure. Biofilm prevents antibiotic delivery to the bacteria. In addition, biofilms act as a reservoir of microorganisms^6^. Therefore, if the initial therapeutic intervention is delayed, then the biofilm formation might engender refractory or relapsing peritonitis and thereby increase the risk of catheter removal and re-development of peritonitis within 1 month after starting treatment. Although the necessary time for biofilm organization on the PD catheter surface remains unclear, animal studies have shown that *Staphylococcus aureus* rapidly formed mature biofilm on wounds within 24 h^8^. Therefore, it might be necessary to start appropriate antibiotic treatment within 24 h of the onset of symptoms, at the latest, for PD-associated peritonitis.

Our results demonstrated that, in addition to ST time, concomitant tunnel infection is independently correlated with PD failure attributable to peritonitis. Several reports have described factors associated with undesirable outcomes in PD related peritonitis. Yang et al. reported that the etiologic source of the infection, concomitant tunnel/exit-site infection, and abdominal visceral catastrophes are likely to be associated with PD catheter loss attributable to peritonitis^9^. Moreover, tunnel infection and exit-site infection are known as major predisposing factors for PD-related peritonitis. Therefore, careful physical and ultrasonographic examination of the catheter tunnel should be performed routinely when peritonitis is diagnosed^10^. Early, prompt diagnosis and management of PD catheter-related infection before development of peritonitis are important to prolong PD catheter survival. In another report, Ram et al. identified hypotension, loose stool, and paralytic ileus as risk factors of catheter loss^11^. These symptoms might reflect systemic or abdominal manifestations of PD-related peritonitis, leading to unstable circulation and malnutrition. Therefore, early start of treatment before developing systemic or abdominal complications is warranted to overcome peritonitis.

In our retrospective cohort, the first peritonitis episode and PD duration were associated with delayed ST time (ST time ≥ 24 h). Although all patients were educated at PD initiation about actions to be taken at the onset of peritonitis, it might be difficult to practice those actions when peritonitis occurs for the first time after a long period. Patients with long PD duration tend to self-determine based on their own experiences. Opportunities for re-education about initial actions to be taken at the onset of peritonitis should be provided for PD patients. Although re-training programs reportedly are unable to prevent the onset of peritonitis^12^, continuous education might contribute to reduction of PD catheter loss after peritonitis. Apparently, hospital accessibility had little effect on delayed ST time because the distance from the location at which patients noticed cloudy effluent to the hospital was not related to ST time ≥ 24 h.

Comparison of the values obtained for variables between groups with ST time < 24 h (early group) and ≥ 24 h (late group) revealed that patients in the late group were more likely to use oral antibiotics before visiting a hospital. Although pre-prescription of oral antibiotics might be useful to avoid treatment delay in the case of difficulty of a prompt visit, it might rather delay visiting and initiation of intraperitoneal antibiotics. No evidence was found for associations between the use of oral antibiotics in advance and reduction of the catheter removal rate. From these perspectives, rigorous education about the appropriate use of oral antibiotics is apparently fundamentally important for PD patients.

Detection of the causative organism is important for appropriate treatment of PD-related peritonitis. However, the rate found for culture negative peritonitis was as high as 30% in our study. A possible reason for that high rate is that the detection sensitivity of the causative organisms might be low because microbiological examination of PD effluent using blood-culture bottle was not performed until 2013. Another reason is that, as described in *Methods*, oral antibiotics were pre-prescribed for peritonitis until 2013. Although patients were educated to take oral antibiotics after draining cloudy PD effluent for sampling, not all patients were able to do so. Oral antibiotics administered before obtaining culture specimens made it difficult to identify causative organisms and to choose the optimal treatment. By correcting those shortcomings above, adopting PD effluent culture using blood-culture bottle and withdrawal of oral antibiotics pre-prescription, the rate of culture-negative peritonitis improved to 12% after 2014 in our hospital.

The present study had some limitations. First, there might be some discrepancy between the actual time and patient-reported time because information about the time patients became aware of the cloudy effluent was obtained from medical record entries based on patient interviews during the emergency visit. Furthermore, we examined only a small number of patients from a single institution. Consequently, a larger cohort study with other facilities must be undertaken to establish predictors associated with catheter removal. Second, because this was a retrospective observational study, the severities of peritonitis between removal and non-removal groups might not be equal, although only one patient died, because of peritonitis. Third, we might not have investigated or collected sufficient data of unknown factors affecting catheter removal.

Despite those limitations, our study presents some clinical implications. In conclusion, patients with initiation of treatment more than 24 h after awareness of abnormal effluent were more likely to require PD catheter removal because of peritonitis. Long PD duration was related to delayed initiation of treatment. Continuous education for patients with long PD duration about prompt visitation at the onset of peritonitis might be necessary to improve outcomes including PD catheter survival. Although further research is needed, our study suggests several hints at better management to overcome PD peritonitis and to achieve longer PD life.

## Materials and methods

### Patients and data collection

This study, conducted at a single center (The University of Tokyo Hospital), was approved by Institutional Review Board of The University of Tokyo (#2879). This study retrospectively collected data from medical records. Therefore, informed consent was waived by Institutional Review Board of the Research Ethics Committee of the Faculty of Medicine of the University of Tokyo. All methods of research were performed in accordance with the Declaration of Helsinki.

We retrospectively collected the medical records of 128 PD-related peritonitis cases for 66 patients who started PD during 2001–2019 at our hospital as their first renal replacement therapy. All PD patients at our hospital had undergone PD therapy using biocompatible neutral pH solution (Terumo Corp., Tokyo, Japan). PD-related peritonitis was diagnosed based on the criteria of ISPD guidelines. Peritonitis because of nontuberculous mycobacteria (NTM, *N* = 1), fungus (*N* = 2), *Pseudomonas aeruginosa* (*N* = 5), and *Serratia *spp*.* (*N* = 3) were excluded from our study because clinical practice at our facility has been immediate catheter removal in cases for these organisms irrespective of treatment duration. Peritonitis caused by all these organisms is thought to be too difficult to cure using antibiotic treatment alone^1, 13^. Two cases of peritonitis for which the outcome of PD catheter survival was not able to be followed-up were excluded. Six cases for which antibiotic treatment was continued 1 month after the onset of peritonitis were also excluded because the influence of diseases other than peritonitis might be a concern. Consequently, a total of 109 peritonitis cases in 62 patients were examined for this study (Fig. 1).

The time from awareness of abnormalities of PD effluent by patients or their families to the start of intraperitoneal or intravenous administration of antibiotics in hospital (Sign to Treatment time; ST time) was calculated based on their medical records. To ensure the certainty of the calculation results, the ST time was calculated from medical records by one nephrologist and one nurse independently. It was classified into two categories: < 24 and ≥ 24 h. When there was a discrepancy in the determination of the ST time category between the two investigators, another nephrologist determined the category after calculating the ST time after reviewing medical records independently. We also identified patients who took oral antibiotics before visiting the hospital. These oral antibiotics were (1) pre-prescribed to be used in case of difficulty in visiting our hospital immediately at the onset of peritonitis or (2) prescribed as the treatment of other diseases. Initial treatment for peritonitis was conducted according to the ISPD guideline^10^. The decision of catheter removal was made by the attending physicians according to two situations: (1) when five days of treatment with appropriate empirical antibiotics was ineffective; (2) when a co-existing tunnel infection extended beyond the subcutaneous cuff. Treatment strategies including selection or change of antibiotics and catheter removal had been decided by attending physicians independently of this study. Patients were followed until 1 month after starting treatment for peritonitis at the hospital.

Clinical and laboratory data, including age, gender, cause of end-stage renal disease, white blood cell count, and results of culture of PD effluent at the start of treatment in hospital were obtained. For PD fluid culture, only a simple sterilized tube was used until 2013; blood-culture bottles have also been available at our hospital since 2014.

## Outcomes

Between ST time < 24 and ≥ 24 h groups, this study compared PD catheter survival rates within 1 month after starting treatment. We examined whether the ST time category was independently associated with catheter removal or death because of PD peritonitis. Clinical factors that were related to delayed start of treatment (ST time ≥ 24 h) were investigated.

### Statistical analysis

All statistical analyses were conducted using software (JMP 14; SAS Institute Inc., Cary, NC). Continuous data were expressed as mean ± standard deviation or median (interquartile range). Student *t*-tests or Mann–Whitney *U*-tests were used to compare continuous variables. The chi-square test or Fisher’s exact test was used to compare categorical variables. The Kaplan–Meier method and the log-rank test were used to compare differences in PD catheter survival or re-development of peritonitis within 1 month after initiation of treatment between groups. Univariate and multivariate logistic regression analyses were used to examine significant factors associated with PD catheter removal. Factors with a *p* value < 0.05 in univariate logistic regression analysis were included in multivariate analysis. Results for which a *p* value was less than 0.05 were inferred as significant.

## Supplementary Information

Supplementary Legends.

Supplementary Figures.

Supplementary Tables.
