# Supplementary material for: Time until treatment initiation is associated with catheter survival in peritoneal dialysis-related peritonitis
Source: Sci Rep. 2021 Mar 22;11:6547. doi: 10.1038/s41598-021-86071-y (PMC7985378; doi:10.1038/s41598-021-86071-y)
Supplement: Supplementary file 1 — Supplementary Legends. [file 41598_2021_86071_MOESM1_ESM.docx]

Supplementary Figure 1.

Kaplan–Meier analysis for the rate of catheter removal and relapse/recurrence of peritonitis during 1 month from initiation of treatment. No significant difference was found in the PD catheter removal rate (A) and a composite outcome of catheter removal or relapse/recurrence of peritonitis (B) between the groups with ST time >= 12 hr and < 12 hr (*p*= .21, *p*= .08, respectively).

Supplementary Figure 2.

No significant difference in the PD catheter removal rate was found between the groups with ST time >= 18 hr and < 18 hr (*p*= .06). (A) Rate of PD catheter survival or re-development of peritonitis within 1 month after initiation of treatment was significantly higher in the ST time >= 18 hr group than that with ST time < 18 hr group (*p*= .01) (B)
