# Supplementary figures and images for: Time until treatment initiation is associated with catheter survival in peritoneal dialysis-related peritonitis
Source: Sci Rep. 2021 Mar 22;11:6547. doi: 10.1038/s41598-021-86071-y (PMC7985378; doi:10.1038/s41598-021-86071-y)

Supplementary figure

Fig. S1

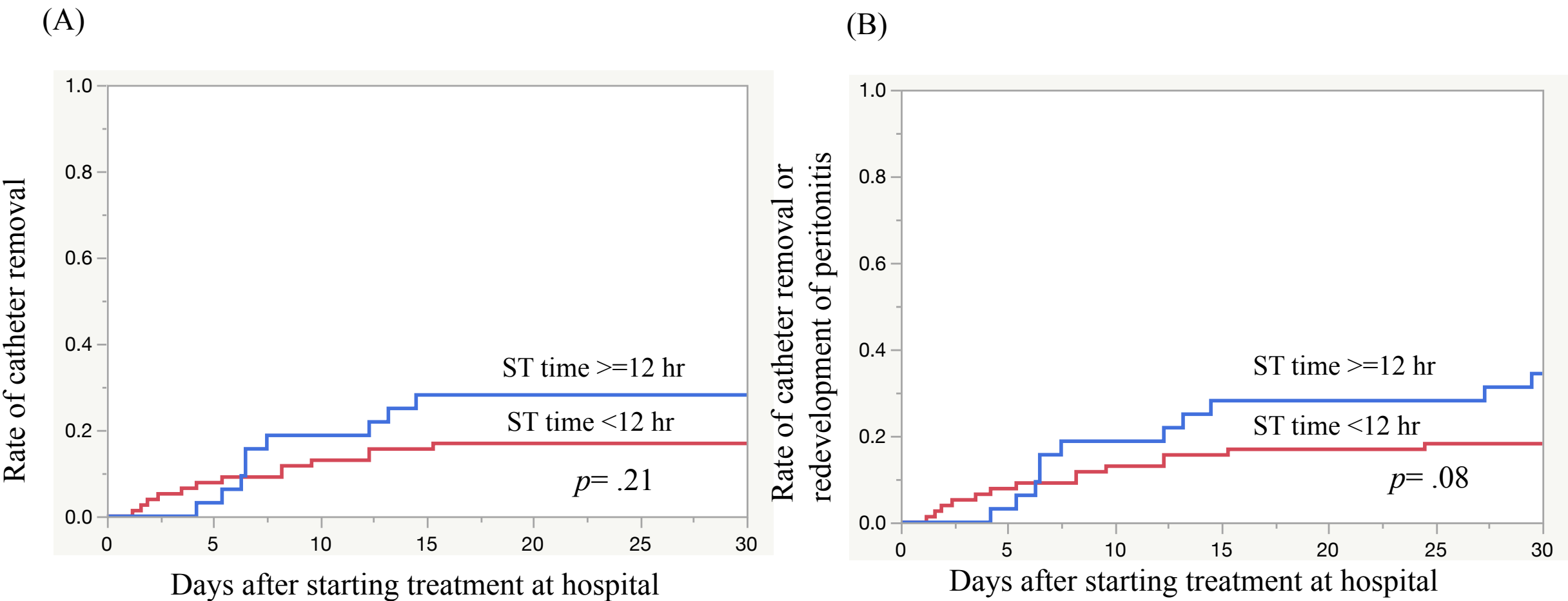

Fig. S2

(A)

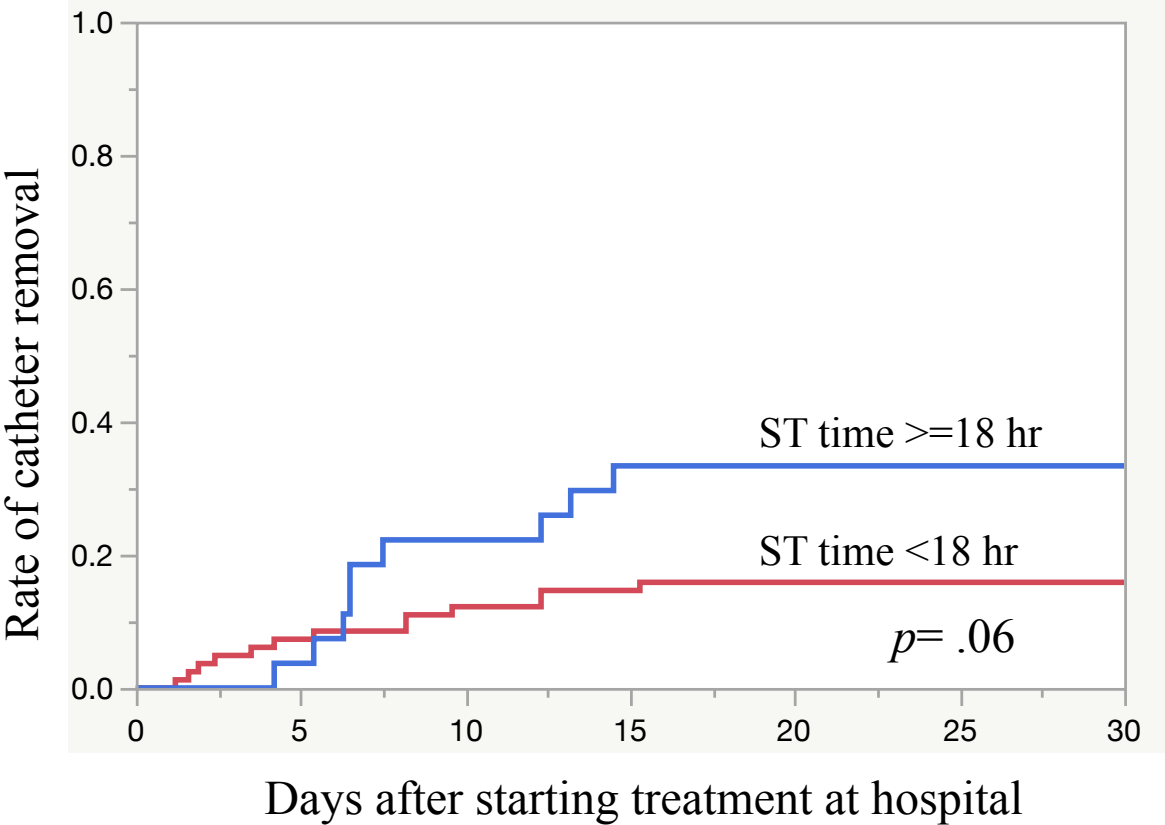

(B)

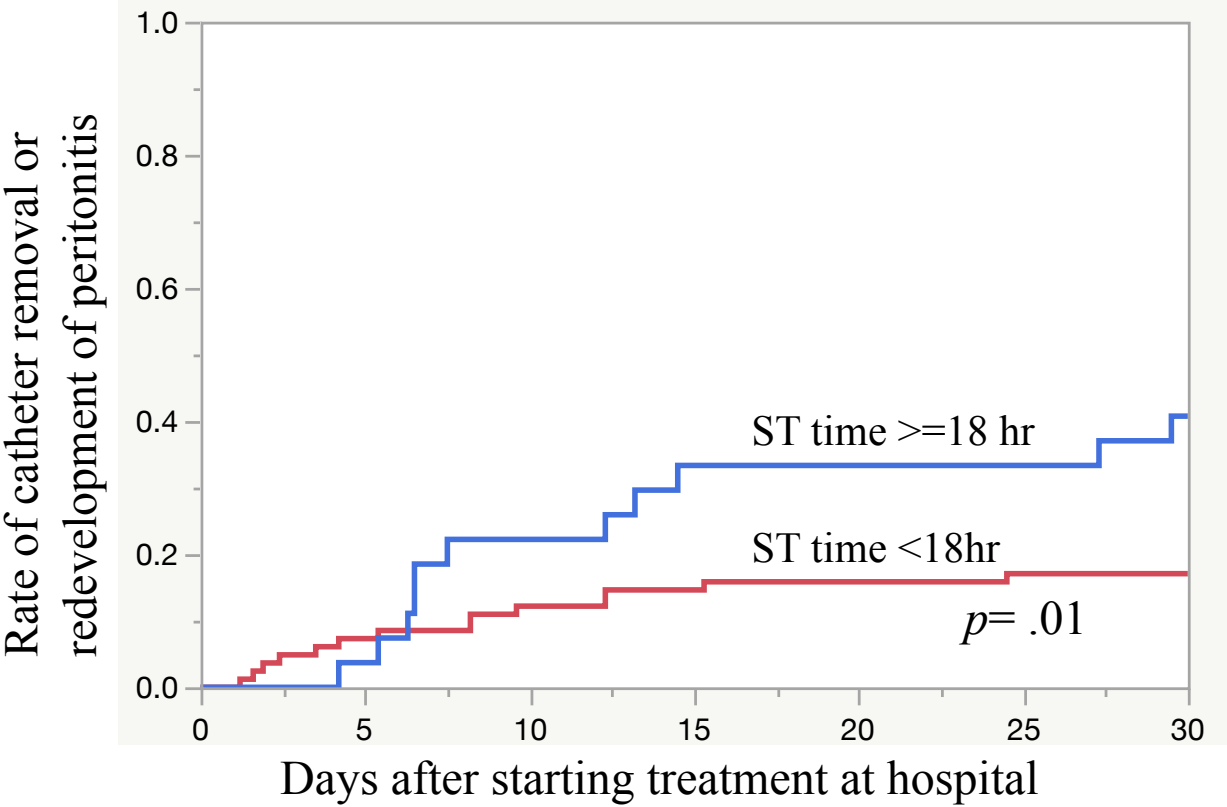

Supplement: Supplementary file 2 — Supplementary Figures. [file 41598_2021_86071_MOESM2_ESM.pdf]
