# Supplementary material for: Time until treatment initiation is associated with catheter survival in peritoneal dialysis-related peritonitis
Source: Sci Rep. 2021 Mar 22;11:6547. doi: 10.1038/s41598-021-86071-y (PMC7985378; doi:10.1038/s41598-021-86071-y)
Supplement: Supplementary file 3 — Supplementary Tables. [file 41598_2021_86071_MOESM3_ESM.pdf]

Time until treatment initiation is associated with catheter survival in peritoneal dialysis-related peritonitis

Rikako Oki[1)] †, Shiho Tsuji[1)] †, Yoshifumi Hamasaki[1)*], Yohei Komaru[1)], Yoshihisa Miyamoto[1)], Ryo Matsuura[1)], Daisuke Yamada[1)], Kent Doi[2)], Haruki Kume[3)], Masaomi Nangaku[1)]

1) Department of Hemodialysis and Apheresis, The University of Tokyo Hospital

2) Department of Acute Medicine, The University of Tokyo Hospital

3) Department of Urology, The University of Tokyo Hospital

†：Rikako Oki and Shiho Tsuji contributed equally to this work.

＊Address correspondence:

Yoshifumi Hamasaki, MD, PhD

Department of Hemodialysis and Apheresis, The University of Tokyo Hospital

Postal address: 7-3-1 Hongo, Bunkyo-ku, Tokyo 113-8655, Japan

E-mail address: yhamasaki-tky@umin.ac.jp

Tel.: +81-3-3815-5411

FAX: +81-3-5800-8806

Supplementary Table 1. Comparison of causative microorganism between removal and non-removal groups

microorganism [*n*(%)]	removal group (*N*=22)	non-removal group (*N*=87)	*p*
CNS	2 (9%)	14 (16%)	.41
Streptococcus spp.	1 (5%)	16 (18%)	.10
Staphylococcus aureus	4 (18%)	6 (7%)	.10
Other gram-positive cocci	3 (14%)	4 (5%)	.12
Gram-negative bacilli*	2 (9%)	12 (14%)	.56
Other bacteria**	0 (0%)	3 (3%)	.38
No growth	10 (45%)	32 (37%)	.46

*: except for *P. aeruginosa* and *Serratia spp.*

**: Corynebacterium, Gram-positive bacilli

Supplementary Table 2. Comparison of causative microorganism between groups of ST time more or less than 24 hr

microorganism [*n*(%)]	ST time<24 hr (*N*=88)	ST time>=24 hr (*N*=21)	*p*
CNS	14 (16%)	2 (10%)	.46
Streptococcus spp.	14 (16%)	3 (14%)	.85
Staphylococcus aureus	8 (9%)	2 (10%)	.95
Other gram-positive cocci	7 (8%)	0 (0%)	.18
Gram-negative bacilli*	10 (11%)	4 (19%)	.34
Other bacteria**	2 (2%)	1 (5%)	.53
Culture negative peritonitis	33 (38%)	9 (43%)	.65

*: except for *P. aeruginosa* and *Serratia spp.*

**: Corynebacterium, Gram-positive bacilli

Supplementary Table 3. Comparison between PD catheter removal and non-removal groups

	all (*N*=109)	removal group (*N*=22)	non-removal group (*N*=87)	*p* (removal vs. non-removal)

ST time >=12 hr [*n*(%)]	32 (29%)	9 (41%)	23 (26%)	.18
ST time >=18 hr [*n*(%)]	27(25%)	9 (41%)	18 (21%)	.05

Supplementary Table 4. Results of univariate logistic regression analysis for PD catheter removal

**Variables**	univariate	
	**OR (95% CI)**	***p***
ST time >=12 hr	1.93 (0.73–5.10)	.19
ST time >=18 hr	2.65 (0.98–7.18)	.06
